# Supplementary figures and images for: Adult-onset temporal lobe epilepsy suspicious for autoimmune pathogenesis: Autoantibody prevalence and clinical correlates
Source: PLoS One. 2020 Oct 29;15(10):e0241289. doi: 10.1371/journal.pone.0241289 (PMC7595292; doi:10.1371/journal.pone.0241289)

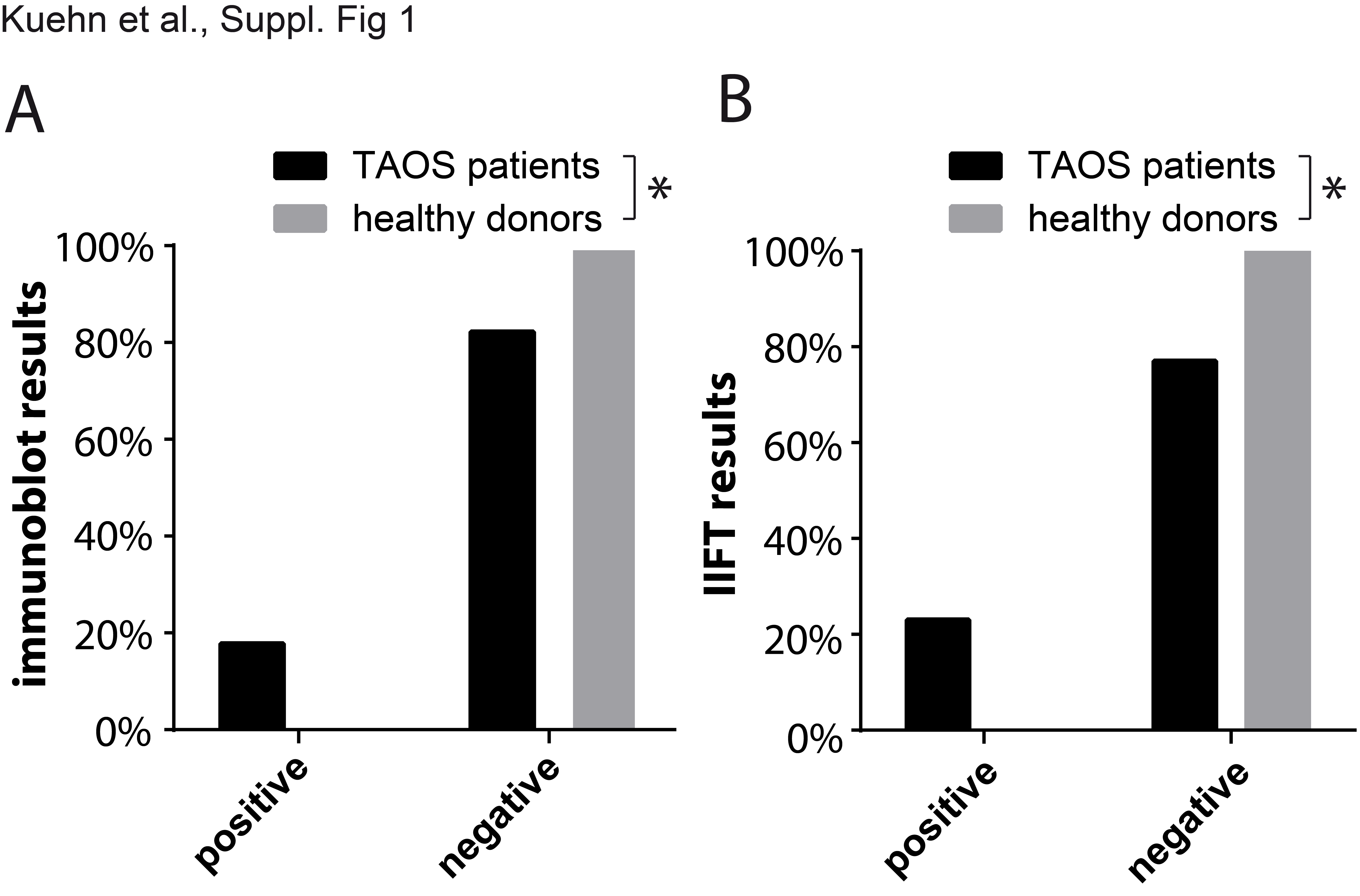

Supplement: S1 Fig — Healthy donors had significantly less positive (A) immunoblot and (B) IIFT results in comparison to the TAOS patient cohort (TAOS patients n = 765, healthy donors n = 27, Chi-square-test group comparison: *p<0.05). (TIF) [file pone.0241289.s001.tif]

Ctr serum (healthy donor)

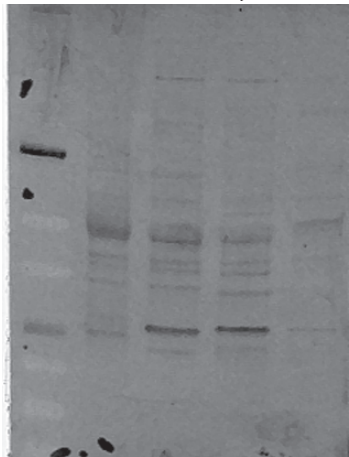

165 - 2016 serum

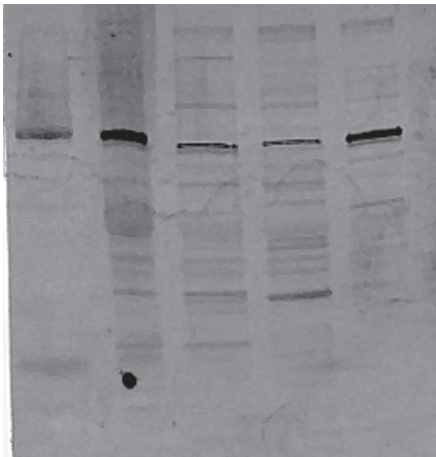

205 - 2016 serum

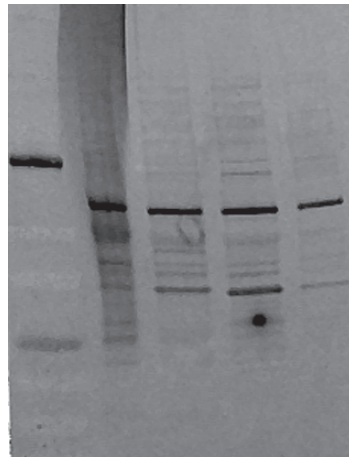

377 - 2016 serum

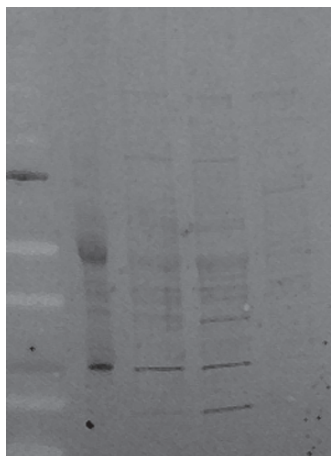

462 - 2015 serum

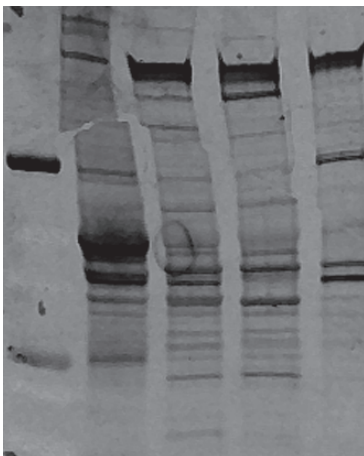

571 - 2015 serum

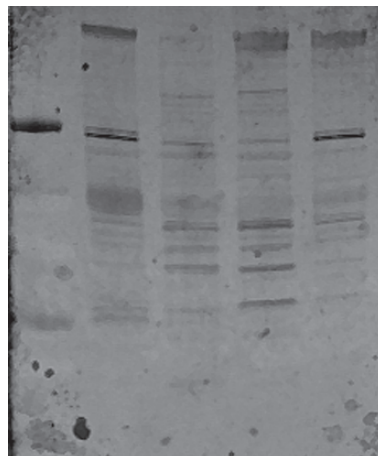

Ctr CSF(healthy donor)

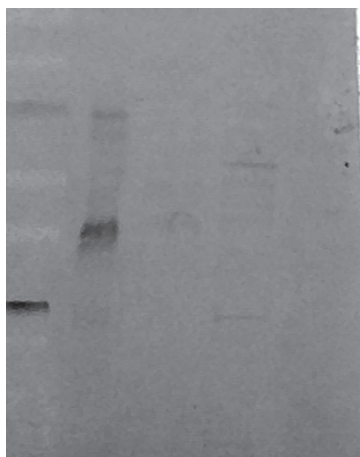

410 - 2015 CSF

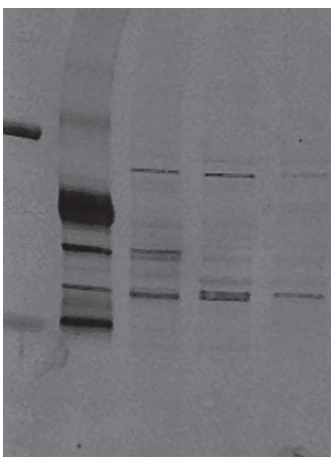

629 - 2015 CSF

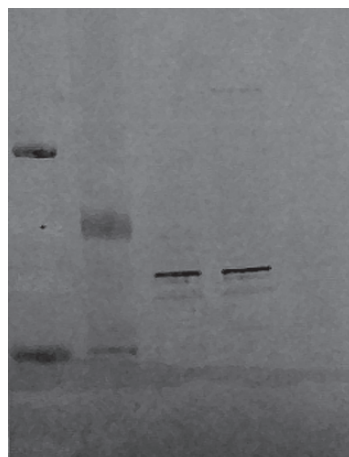

166 - 2016 CSF

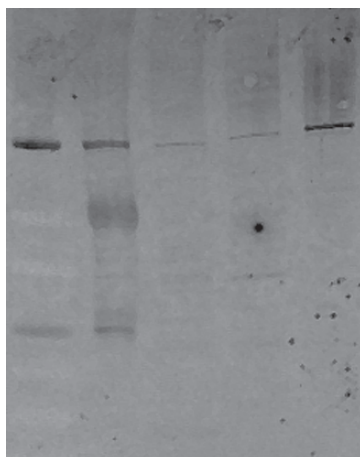

206 - 2016 CSF

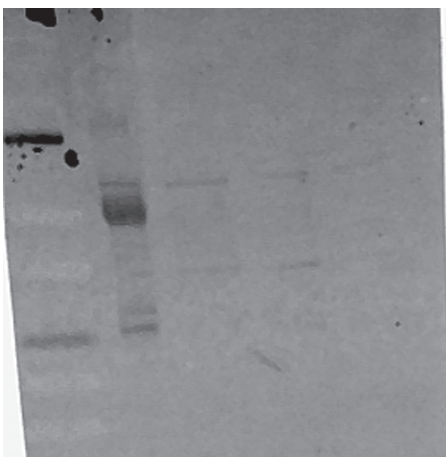

378 - 2016 CSF

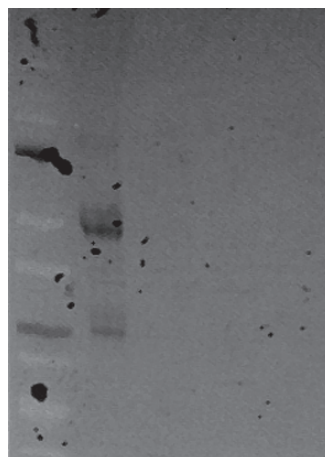

Supplement: S3 File — (PDF) [file pone.0241289.s004.pdf]

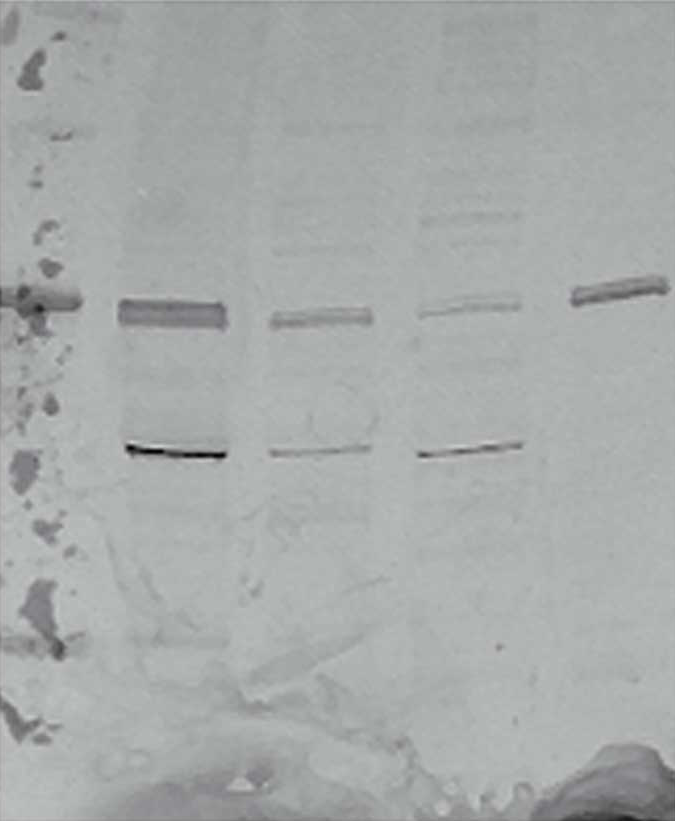

Supplement: S1 Raw image — (TIF) [file pone.0241289.s005.tif]
