# Supplementary material for: Adult-onset temporal lobe epilepsy suspicious for autoimmune pathogenesis: Autoantibody prevalence and clinical correlates
Source: PLoS One. 2020 Oct 29;15(10):e0241289. doi: 10.1371/journal.pone.0241289 (PMC7595292; doi:10.1371/journal.pone.0241289)
Supplement: S1 File — (DOCX) [file pone.0241289.s002.docx]

**Supplemental Methods**

**Preparation of lysate and synaptosomes for immunoblotting.** For lysate preparation 2 ml of lysis buffer [0.32 M sucrose pH 7.4, 100 µM Ethylenediaminetetraacetic acid (EDTA), 5 mM 4-(2-hydroxyethyl)-1-piperazineethanesulfonic acid (HEPES), protease inhibitor] were added per 1 g of whole mouse/rat brain or hippocampal human tissue. Tissue was homogenized on ice using a Polytron PT 1200E dispersing aggregate until suspension was homogenous. The suspension was centrifuged for 1 h at 4°C at 12500 RCF. Supernatants containing the proteins were collected, protein concentration was measured, and samples were stored at -80°C until usage. For synaptosome preparation 6 ml of lysis buffer was added to one mouse brain. Brain tissue was homogenized using a Potter S Homogenizer (Braun Biotech International) at 900 rpm for 7 times. The suspension centrifuged for 15 min at 4°C at 3000 RCF. Supernatants were collected and centrifuged for 25 min at 4°C at 12500 RCF. The supernatant was discarded, and the pellet resuspended in 300 µl of buffer. Protein concentration was measured, and aliquots were stored at -80°C until usage.

**Immunohistochemistry with human serum or CSF on brain sections.** Human hippocampus, rat cerebrum and cerebellum slices (Microm HM 560 cryostat; Thermo Scientific) were incubated for 2 h at 37°C with blocking buffer (10% FCS + 1% normal goat serum (NGS) in PBS). Blocking buffer was replaced by 100 µl undiluted serum or CSF. As a positive control an antibody against neuronal nuclei (anti-NeuN) was used diluted 1:100 in PBS. Serum/CSF or antibody solution was incubated over night at room temperature in a humidity chamber. After incubation slices were washed two times with PBS each for 5 min. 100 µl of anti-human-biotin antibody (1:500) respective anti-mouse-biotin antibody (1:100) was put on the slice and incubated 2 h at 37°C in a humidity chamber. Slices were washed twice with PBS 5 min each. Binding of antibodies was visualized performing a 3,3′-Diaminobenzidin (DAB)-staining according to manufacturer’s protocol. Samples were additionally stained with H.E. (Hematoxylin and eosin) and covered with a cover slip.
